# Supplementary material for: Analysis of PPARγ Signaling Activity in Psoriasis
Source: Int J Mol Sci. 2021 Aug 10;22(16):8603. doi: 10.3390/ijms22168603 (PMC8395241; doi:10.3390/ijms22168603)
Supplement: Supplementary file 1 [file ijms-22-08603-s001.zip › Supplemental materials_Analysis of PPARg signaling activity in psoriasis/Pathway models/Models images and html files/PPARG inhibits phenotypes assotiated with psoriasis/244961.html]

endothelial cell dysfunction


# Disease endothelial cell dysfunction

|  |  |
| --- | --- |
| URN | urn:agi-disease:endothelial%20cell%20dysfunction |
| Total Entities | 0 |
| Connectivity | 4299 |
| Name | endothelial cell dysfunction |

---

|  |  |
| --- | --- |
| Pathway | PPARG inhibits phenotypes assotiated with psoriasis |
|  | efects selected |
|  | Neighbors of endothelial cell dysfunction |

---

|  |  |
| --- | --- |
| MedScan ID | 9017371 |

---

|  |  |
| --- | --- |
| Alias | endothelial dysfunctions |
|  | diseases of the endothelium |
|  | endothelial disease |
|  | endothelial cell dysfunction |
|  | endothelial cell dysfunctions |
|  | endothelial disorders |
|  | endothelium diseases |
|  | endothelial disorder |
|  | endothelium dysfunction |
|  | endothelium dysfunctions |
|  | dysfunction of endothelium |
|  | endothelial diseases |
|  | endothelial dysfunction |
|  | endothelium disorder |
|  | endothelium disorders |
|  | endothelium disease |
|  | dysfunctions of endothelium |
|  | disease of the endothelium |

---
